# Impact of nanocarrier aggregation on EPR-mediated tumor targeting

**DOI:** 10.3389/fbioe.2023.1222693

**Published:** 2023-07-21

**Authors:** S. P. Surya Teja, N. Damodharan, T. Tamilanban, Vetriselvan Subramaniyan, V. Chitra, Suresh V. Chinni, Ling Shing Wong, Neeraj Kumar Fuloria, Mahendran Sekar, Shivkanya Fuloria, Gobinath Ramachawolran, Siddharthan Selvaraj

**Affiliations:** ^1^ Department of Pharmaceutics, SRM College of Pharmacy, SRM Institute of Science and Technology, Kattankulathur, Tamil Nadu, India; ^2^ Department of Pharmacology, SRM College of Pharmacy, SRM Institute of Science and Technology, Chennai, Tamil Nadu, India; ^3^ Jeffrey Cheah School of Medicine and Health Sciences, Monash University, Bandar Sunway, Malaysia; ^4^ Department of Pharmacology, Center for Transdisciplinary Research, Saveetha Dental College, Saveetha Institute of Medical and Technical Sciences, Saveetha University, Chennai, Tamil Nadu, India; ^5^ Department of Biochemistry, Faculty of Medicine, Bioscience, and Nursing, MAHSA University, Jenjarom, Malaysia; ^6^ Department of Periodontics, Saveetha Dental College and Hospitals, Saveetha Institute of Medical and Technical Sciences, Saveetha University, Chennai, Tamil Nadu, India; ^7^ Faculty of Health and Life Sciences, INTI International University, Nilai, Malaysia; ^8^ Faculty of Pharmacy, AIMST University, Bedong, Malaysia; ^9^ School of Pharmacy, Monash University Malaysia, Subang Jaya, Selangor, Malaysia; ^10^ Department of Foundation, RCSI and UCD Malaysia Campus, Pinang, Malaysia; ^11^ Faculty of Dentistry, AIMST University, Bedong, Malaysia

**Keywords:** biodistribution, chitosan, lyophilization, cancer treatment, nanocomposite, targeted tumor drug

## Abstract

The aim of this study was to investigate the influence of excipients on retaining the particle size of methotrexate (MTX) loaded chitosan nanocarriers (CsNP) during lyophilization, which relates to the ability to enlarge the particle size and target specific areas. The nanocarriers were prepared using the ionic gelation technique with tripolyphosphate as a crosslinker. Three lyophilized formulations were used: nanosuspension without Lyoprotectant (NF), with mannitol (NFM), and with sucrose (NFS). The lyophilized powder intended for injection (PI) was examined to assess changes in particle size, product integrity, and comparative biodistribution studies to evaluate targeting ability. After lyophilization, NFS was excluded from *in-vivo* studies due to the product melt-back phenomenon. The particle size of the NF lyophile significantly increased from 176 nm to 261 nm. In contrast, NFM restricted the nanocarrier size to 194 nm and exhibited excellent cake properties. FTIR, XRD, and SEM analysis revealed the transformation of mannitol into a stable β, δ polymorphic form. Biodistribution studies showed that the nanocarriers significantly increased MTX accumulation in tumor tissue (NF = 2.04 ± 0.27; NFM = 2.73 ± 0.19) compared to the marketed PI (1.45 ± 0.25 μg), but this effect was highly dependent on the particle size. Incorporating mannitol yielded positive results in restricting particle size and favoring successful tumor targeting. This study demonstrates the potential of chitosan nanocarriers as promising candidates for targeted tumor drug delivery and cancer treatment.

## Highlights

1. The study aimed to investigate how different excipients affect the particle size of methotrexate-loaded chitosan nanocarriers during lyophilization. The ability to control particle size is crucial for targeting specific areas.
2. The addition of mannitol as a lyoprotectant (NFM formulation) restricted the nanocarrier size to 194 nm and demonstrated excellent cake properties. The transformation of mannitol into a stable β, δ polymorphic form was observed through FTIR, XRD, and SEM analysis.
3. Biodistribution studies showed that the nanocarriers significantly increased methotrexate (MTX) accumulation in tumor tissue compared to the marketed injection. The formulation with mannitol (NFM) exhibited improved tumor targeting (2.73 ± 0.19 µg) compared to the nanosuspension without lyoprotectant (NF, 2.04 ± 0.27 µg).
4. The study demonstrates the potential of chitosan nanocarriers as promising candidates for targeted tumor drug delivery. The results highlight the importance of controlling particle size and the effectiveness of incorporating mannitol as an excipient.

## Introduction

Nanocarrier-mediated cancer targeting, capitalizing on the Enhanced Permeation and Retention (EPR) effect, has gained attention in recent years (Golombek et al., 2018). Physiological features such as fenestrated blood vessels, a low pH value, and the hypoxic environment of tumor tissue are also helpful in designing environment-responsive nanocarriers. Investigations indicate that the cancer vasculature has a pore size ranging from 200 to 1200 nm, and the pH of cancer tissue is around 5.5 in its cytoplasm (Krock et al., 2011). Nevertheless, complications related to the production and stability of nanocarriers outweigh their targeting effectiveness (Din et al., 2017). Besides the difficulty in the robust preparation of nanocarriers, maintaining the particle size of nanoformulations and product storage is also challenging (Pérez-Herrero and Fernández-Medarde, 2015).

In this regard, lyophilization is widely used as a drying technique for aqueous nanoformulations to improve their stability and shelf life (Bhat et al., 2023). The advantages of nanocarrier lyophilization outweigh its demerits, despite it being an expensive and time-consuming process. However, due to its highly energy-intensive nature and the application of stressful pressure and temperature, lyophilization can cause particle aggregation and destabilization effects (Ngamcherdtrakul et al., 2018). Additionally, failures in retaining the physical, chemical, and pharmaceutical properties of the initial liquid formulation have also been observed (Ball et al., 2016; Ngamcherdtrakul et al., 2018). To address these issues, lyoprotectants, inert substances added to the aqueous formulation during lyophilization, are often employed to prevent aggregation and disruption of nanocarriers (Guimarães et al., 2019).

The current study was based on the hypothesis that nanocarriers with a particle size of less than 200 nm can easily penetrate the tumor vasculature due to the EPR effect, allowing for passive targeting (Teja and Damodharan, 2018). Additionally, incorporating nanocarriers with a pH-dependent polymer can leverage the active targeting of tumor cells, facilitating the release of drugs inside cancerous cells. Chitosan, selected for its pH-responsive behavior and exceptional biocompatibility, was chosen as the polymer for preparing the nanocarriers (Li et al., 2018; Pilipenko et al., 2019). Meanwhile, methotrexate (MTX), a folic acid analogue widely used in the treatment of neck and colon cancers (Jang et al., 2019), was incorporated into the chitosan nanocarriers (CsNP).

The current study was designed to evaluate the ability of mannitol and sucrose in maintaining the desired particle size in lyophilized powder for injection, as the particle size of nanocarriers is crucial for successful EPR targeting. Additionally, the study aimed to assess the dual targeting ability of pH-responsive methotrexate-loaded chitosan nanocarriers.

## Materials and methods

### Materials

Aeon Formulations Pvt Ltd. in Puducherry and SRM Research Institute provided Methotrexate and Methotrexate Powder for injection, respectively. Low molecular weight (LMW) chitosan, tripolyphosphate sodium, sodium hydroxide, and glacial acetic acid were purchased from Sigma-Aldrich Chemical Co. Ltd. HPLC-grade analytical reagents were used. Animals were purchased from Sri Venkateshwara Breeders after obtaining IEAC approval.

### Preparation and lyophilization of nanocarriers

A methotrexate-loaded chitosan nanocarrier with a predetermined size of 180 nm was prepared by following the optimized formulation determined in our previous research (Teja and Damodharan, 2018). In brief, methotrexate and tripolyphosphate, the cross-linking agents, were dissolved together in distilled water. The former solution was then added dropwise to the chitosan solution, previously dissolved in 1% acetic acid. The process was carried out at 800 RPM at room temperature for 30 min to facilitate the ionic gelation between the cationic chitosan and the anionic methotrexate and tripolyphosphate. The resulting nanosuspension underwent cycles of centrifugation to remove residual acetic acid, and typical characterization studies were performed (Teja and Damodharan, 2018).

To protect the nanoformulation during the freeze-drying process, mannitol (10% w/v) and sucrose (10% w/v) were added as lyoprotectants. Three types of formulations were prepared: the nanoformulation without lyoprotectant (NF), the nanoformulation with mannitol (NFM), and the nanoformulation with sucrose (NFS). These formulations were filled into glass vials for freeze-drying. The samples were pre-chilled at −60°C for 24 h and then lyophilized using a Lark Inc. lyophilizer in India, with a condenser temperature of −70°C and a pressure of 0.1 Pa. The process continued until the samples were free of moisture, and the vials were sealed with rubber closures after completion of the process (Duru et al., 2015; Yap et al., 2021), resulting in the preparation of a powder for injection (PI).

### Characterization of lyophile

The freeze-dried lyophiles were analyzed using Fourier transmission infrared spectroscopy (FTIR) with a BRUKER spectrometer (ALPHA FT-IR) operating in the range of 4000 cm−1 to 400 cm−1. X-ray diffraction (XRD) analysis was conducted using an X'Pert3 MRD (XL) instrument operated at 30 kV and 15 mA to assess any stress effects caused by the lyophilization process. Additionally, the particle size of the lyophiles was determined using a Horiba Zetasizer (SZ-100 nanoparticle) to evaluate the impact of lyophilization on size and aggregation (Romero-Torres et al., 2007; Yap et al., 2021).

### Scanning electron microscopy

The surface morphology of the freeze-dried lyophiles was examined using scanning electron microscopy (SEM) to conduct a topographical comparison. The SEM analysis was performed using a Quanta FEG instrument operated at 15–30 kV in high vacuum mode. The lyophiles were spread over an aluminum stub, and a high-energy electron beam was directed onto the sample surface in the aforementioned voltage range and vacuum mode. The image obtained from the scattered electrons was assessed to examine the topography of the lyophiles at the microscopic level (Guzzinati et al., 2018; Yap et al., 2021).

### Drug release studies

The lyophilized formulations were packed into a dialysis bag with a molecular cutoff of 12000 Daltons. The bag was then placed in a USP Dissolution apparatus II and subjected to dissolution testing under sink conditions at a temperature of 37°C ± 0.5°C. To evaluate pH-mediated drug release, the dissolution was conducted for 36 h in phosphate buffer at pH 5.8 and pH 7.2, simulating the pH conditions of cancerous and healthy tissues, respectively (Krock et al., 2011). At regular intervals, 5 mL of the sample was withdrawn and replaced with the same volume. The triplicate samples were subsequently analyzed using spectrophotometry at a wavelength of 303 nm to calculate the drug release (Ravi Kumar, 2000).

### Drug release kinetics

The dissolution kinetics were presented as a function of MTX released from the nanocarriers over time. The percentage dissolution at specific time intervals was approximated using established empirical models, and the correlation of the kinetic curve was described for dissolution at pH 5.8 and pH 7.2 (Sreekumar et al., 2018).

### Product evaluation and contamination analysis

The moisture content of the lyophiles was evaluated using Karl Fischer titration. The standard evaluation tests for water for injection were conducted following the prescribed protocols (Patel et al., 2009). As per our in-house policy, the formulations underwent testing for the presence of metallic contaminants prior to *in-vivo* studies. Elemental composition analysis was performed using high-resolution scanning electron microscopy (FEI Quanta FEG 200) equipped with energy dispersive spectroscopy (EDS). The lyophilized formulation was spread onto a specimen stub, and a high-energy electron beam was directed to the zone of interest. The X-ray emission generated was collected by detectors to provide elemental data of the nanoparticles (Scimeca et al., 2018).

### Stability studies

The powder formulations were sealed in amber-colored glass vials and exposed to the relevant climatic conditions based on the ICH climatic considerations for India (Zone IVb). This involved subjecting the formulations to a temperature of 40°C ± 2°C and a relative humidity of 75% ± 5% for a duration of 6 months (Hafner et al., 2011). Samples were withdrawn at the end of the 90th and 180th days, and conventional nano-carrier analysis tests were conducted, including the measurement of particle size, polydispersity index (PDI), and zeta potential.

### Biodistribution studies

Biodistribution studies were conducted in C57BL/6 mice induced with lung tumors (Gangjun et al., 2010). A549 cell lines (5 × 106 cells) were injected into the mice’s flank region, and tumor development was monitored for 30 days. The animals were then divided equally into three groups: Group I received NF, Group II received NFM, and Group III received Marketed MTX powder for injection. Once tumor growth in the lungs was confirmed, a single dose of PI equivalent to 4 mg/kg was reconstituted in water for injection and administered via the tail vein. After 24 h, the mice were sacrificed, and their lungs, spleens, kidneys, and hearts were excised. The excised organs were homogenized, and the amount of MTX present in each organ was analyzed (Ait Bachir et al., 2018). MTX in specific organs was extracted by vortexing in acetonitrile, followed by partitioning in a chloroform layer. The quantification of MTX was performed using HPLC with a Disodium phosphate buffer: Acetonitrile ratio of 89:11 on an Agilent 1260 Infinity Capillary LC system with a Phenomenex RP18 column (Subramaniyan et al., 2022).

### Statistical analysis

Data was represented as mean ± SD. For biodistribution study, ANOVA was used to compare MTX accumulation among the groups. Pair-wise mean comparisons were assessed by Tukey’s *post hoc* analysis. All the results were analyzed at 95% confidence interval and *p* < 0.05 was considered significant.

## Results

### Lyophilization and particle size

**FIGURE 1:**
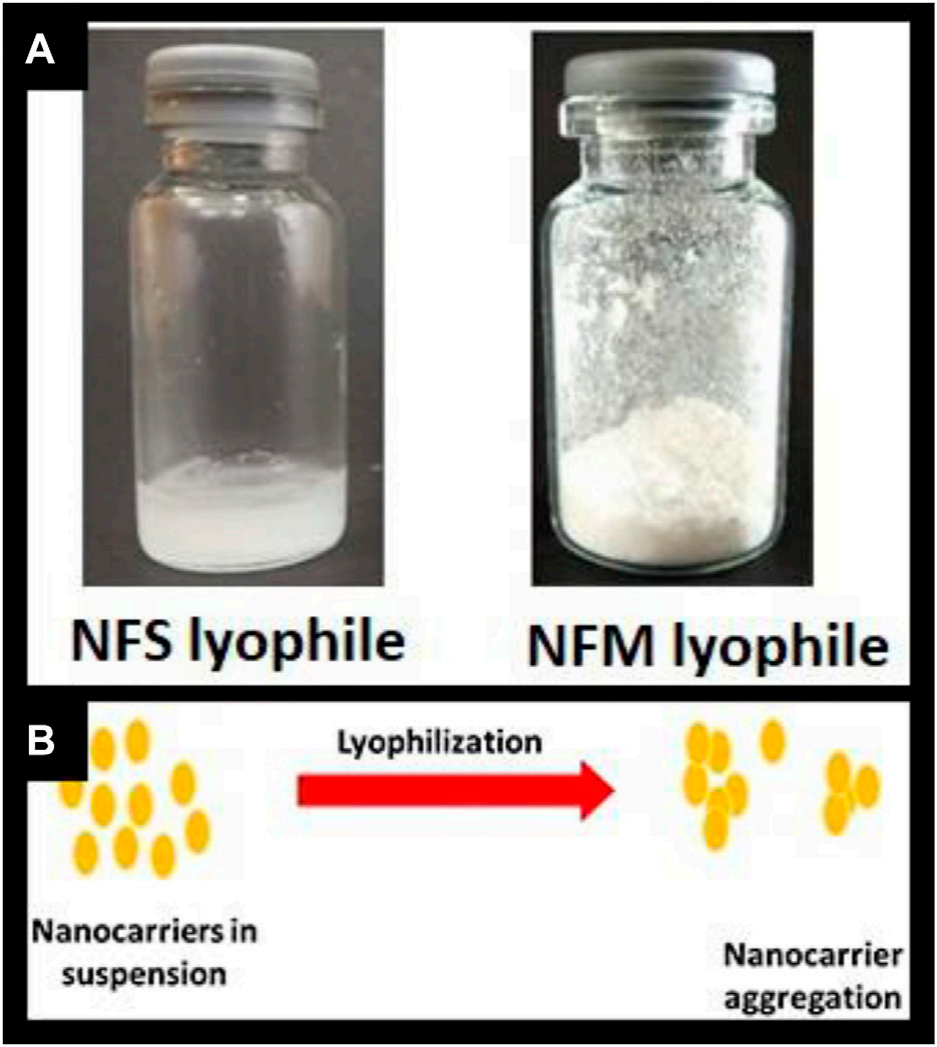
**(A)** Freeze dried product; Figure 1 **(B)** Illustration of nanocarrier aggregation.

The formulation was prepared based on the results of our earlier research using a computational approach, resulting in a particle size of 176 nm. Similar to our previous research, the batch was prepared with MTX concentration of 45 mg.Characterization studies were conducted, and the findings are presented in Table 1. Upon complete freeze-drying of NF, a self-supporting cake with noticeable shrinkage was observed. The mean particle size of NF significantly increased from 176 nm to 261 nm after lyophilization. The dispersity index indicated the formation of nanocarrier aggregation and revealed a large size distribution. During the lyophilization of NFS, fracture propagation was observed in a trans-granular manner within the frozen mass. However, upon completion of the process, NFS did not yield a pharmaceutically elegant product and resulted in product melt-back during storage (Figure 1A). On the other hand, NFM exhibited excellent cake properties (Figure 1A) with an average particle size of 194 nm (Table 2).

**TABLE 1:** Characterization of prepared nanoformulation.

Parameter	Value
Loading Efficiency	10.13%
Entrapment Efficiency	92.18% ± 0.13%
Particle Size	176 ± 4 nm
Polydispersity index	0.41 ± 0.15
Zeta potential	+38.13mV **±** 1.08

**TABLE 2:** Particle size comparison after lyophilization.

Type of product	Particle size	PDI
Nano formulation Before lyophilization	176 ±4nm	0.41
Nanoformulation without lyoprotectant (NF)	261 ±7nm	0.78
Nanoformulation with mannitol (NFM)	194 ±2nm	0.48

PDI: poly dispersity index.

### Characterization and topographical evaluation of lyophile

**FIGURE 2:**
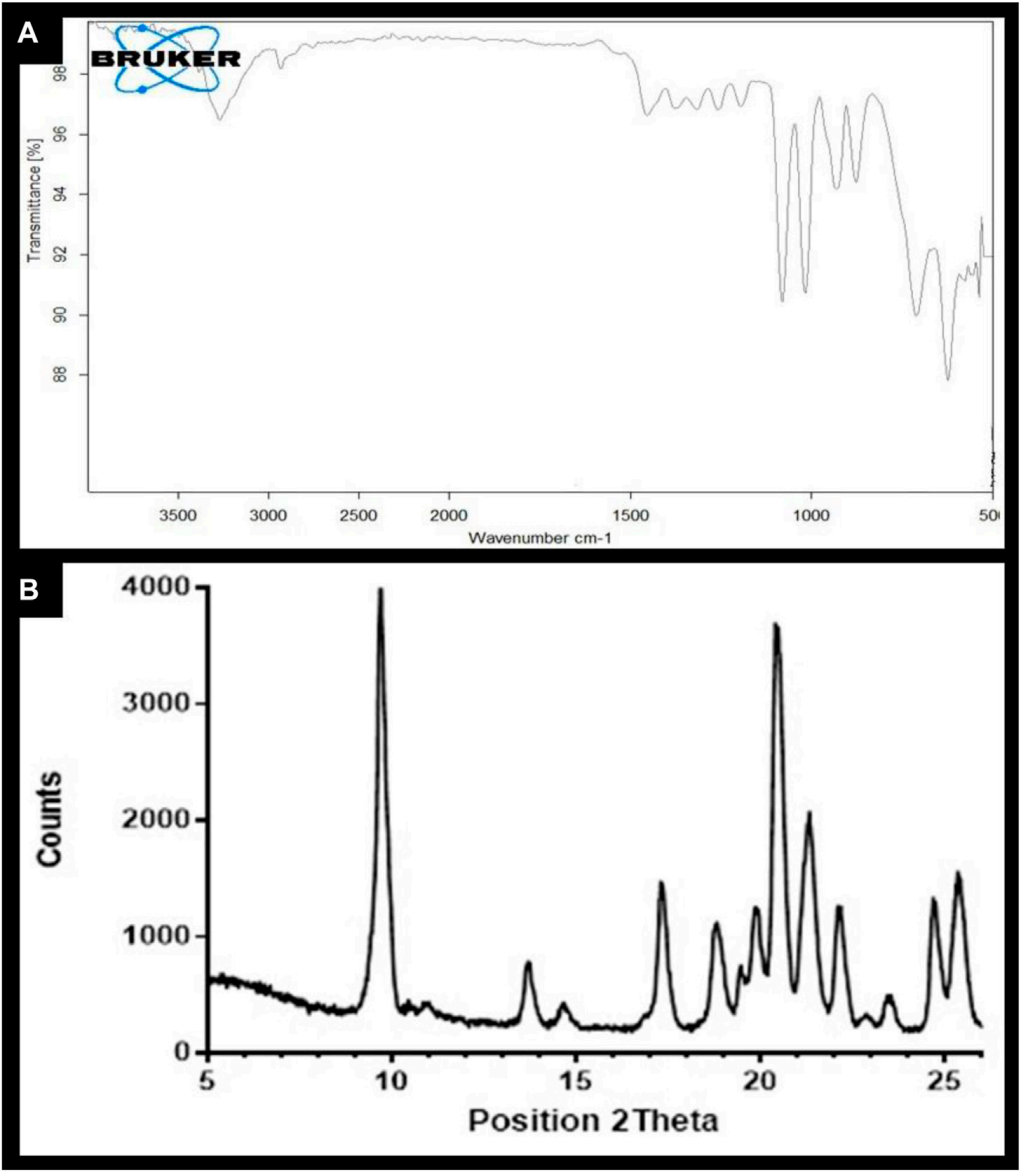
**(A)** FTIR Spectra of NFM lyophile; **(B)** XRD spectra of NFM lyophile.

The FTIR spectra of NF indicated stretching and vibrational peaks at 1265 cm^-1^, 3389 cm^-1^, 1631 cm^-1^, and 1496 cm^-1^, corresponding to P=O, –NH, C=O, and–NH2, respectively. These peaks substantially indicated the presence of methotrexate functional groups and TPP crosslinking (Figure 2). Similarly, the FTIR spectra of NFM revealed the retention of core functional groups of the API, similar to NF. The transmittance pattern in the fingerprint region resembled that of β-mannitol, with high-intensity bending vibrations observed at 1098 cm^-1^, 1003 cm^-1^, 930 cm^-1^, and 623 cm^-1^. Furthermore, vibrations at 1113 cm^-1^ and 876 cm^-1^ resembled the presence of δ-mannitol in the formulation. A sharp and intense peak at a 2θ angle of 9.70, pertaining to δ-mannitol, was also observed (Figure 2). Additionally, a diffraction peak representing β-mannitol was noticed at 14.6°.

**FIGURE 3:**
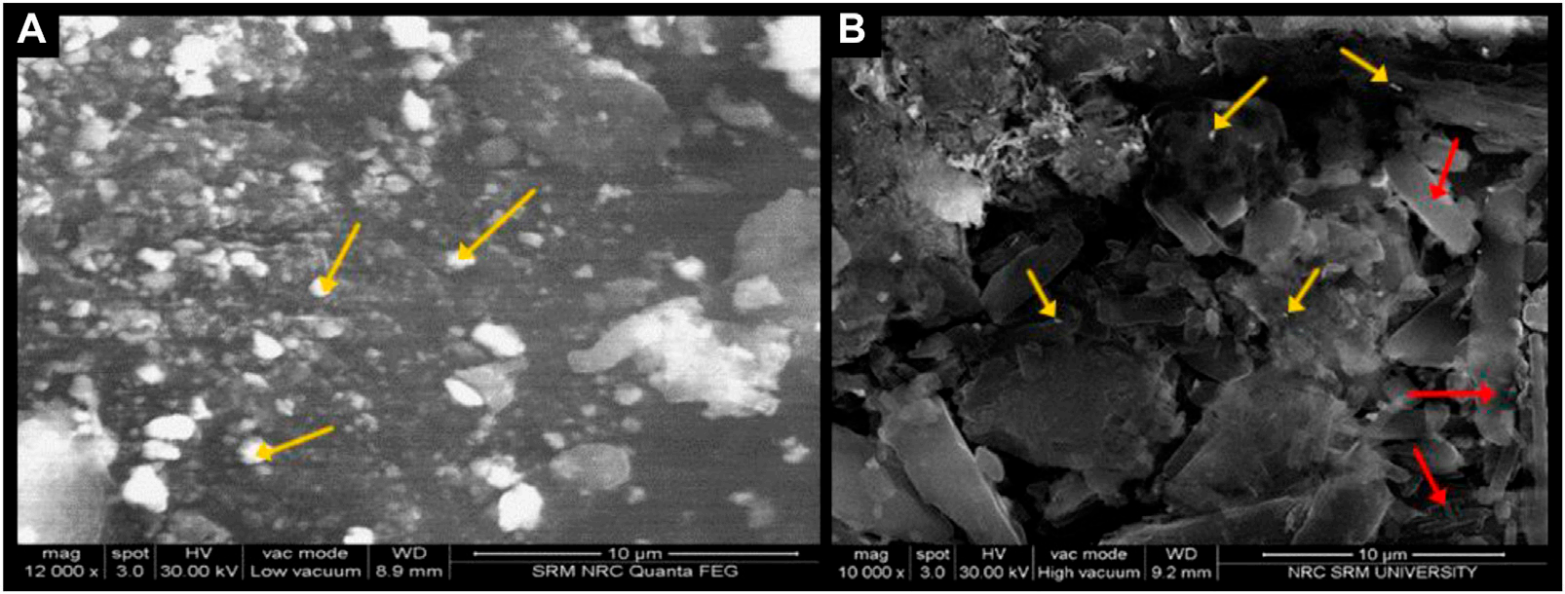
**(A)** Scanning Electron Microscopy image of NF lyophile; **(B)** Scanning Electron Microscopy image of NFM lyophile.

The topography of NF, studied through SEM, featured enlarged nanocarriers and aggregates (Figure 3A). In contrast, reduced aggregation was clearly observed in the NFM lyophile (Figure 3B), displaying morphological features of β and δ-mannitol as thin rectangular sheets.

### Dissolution and drug release kinetics

**FIGURE 4:**
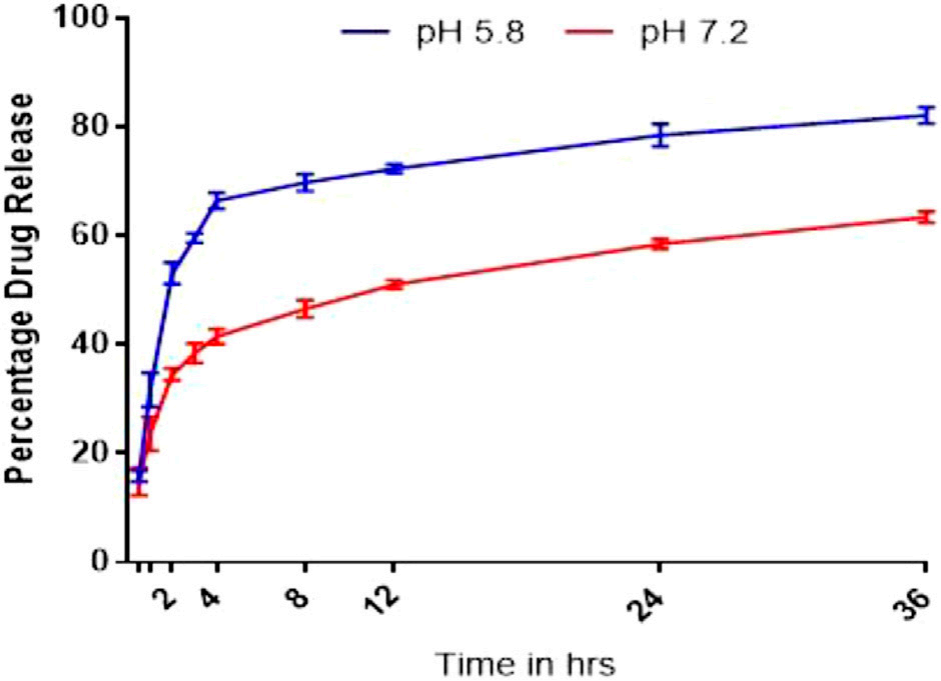
Dissolution profile of NFM lyophile.

Drug release of NF and NFM exhibited a similar biphasic release pattern, characterized by an initial burst release followed by prolonged drug release (Figure 4). Both formulations showed higher drug release in the acidic environment (pH PBS 5.8) compared to pH 7.2. Based on the best fit and higher *R* ^2^ value (*R* ^2^ = 0.960), it was evident that the drug release pattern of NFM was best explained by the Korsmeyer-peppas model. The n-values, n = 0.87 at pH 5.8 and n = 0.67 at pH 7.2, indicated a non-fickian type of drug release (Table 3). A similar drug release behavior was observed with the NF lyophile.

**TABLE 3:** Drug release Kinetics of NFM.

Model	*R* ^2^ value at drug dissolution at pH 5.8	*R* ^2^ value at drug dissolution at pH 7.2
Zero order	0.6748	0.6863
First order	0.7724	0.7546
Hixon crowell	0.7405	0.7321
Higuchi	0.8204	0.8828
Korsmeyer-peppas	**0.9931 n = 0.87**	**0.9967 n = 0.61**

### Product evaluation and contamination analysis

**FIGURE 5:**
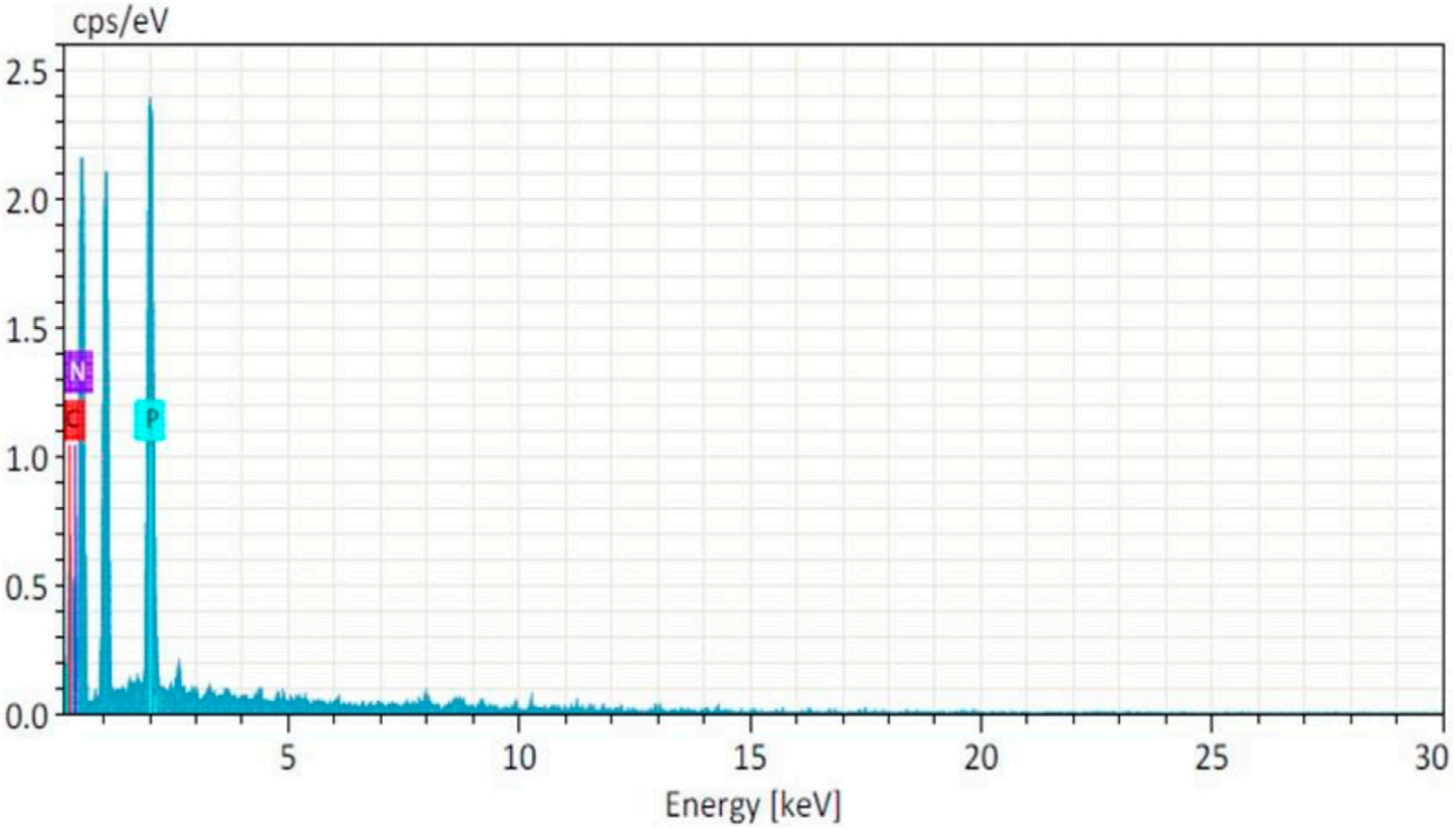
Energy Dispersive spectrum of NFM lyophile.

The moisture content in the NF and NFM lyophiles was determined using the Karl-Fisher titration method and found to be 0.9% and 0.87%, respectively. Other evaluation tests confirmed that the lyophiles met the quality control standards for water for injection (Table 4). EDS studies revealed the absence of heavy metals or toxic elements in the lyophiles, with only the integral components of the ingredients, namely, carbon, nitrogen, and phosphorus, detected at 0.277 KeV, 0.392 KeV, and 2.013 KeV, respectively (Figure 5). Samples were taken at the 90th day and 180th day from the stability chamber and analyzed for nano carrier quality assessment tests, with the results presented in Table 5. No significant difference in quality was observed during the study period.

**TABLE 4:** Evaluation of lyophiles.

Sl. No.	Parameter	NF	NFM
1	Description	Pale Yellow powder for injection	White colored powder for injection
2	Moisture content (%w/w)	0.9% ± 0.02%	0.87% ± 0.03%
3	Reconstitution Time (Sec)	169 ± 4	63 ± 3
4	Reconstituted Solution	Free from visible particles	Clear and free from visible particles

NF , nanoformulation without lyoprotectant; NFM , nanoformulation with mannitol.

**TABLE 5:** Stability studies.

Parameter	Nanoformulation without lyoprotectant (NF)			Nanoformulation with mannitol (NFM)		
	0^th^ day	90th day	180th day	0^th^ day	90th day	180th day
Particle size (nm)	261 ± 3	263 ± 8	259 ± 3	194 ± 4	195 ± 3	195 ± 7
PDI	0.78	0.76	0.76	0.48	0.47	0.48
Zeta Potential (mV)	+38.13 ± 0.3	+37.43 ± 0.5	+37.90 ± 0.6	+36.13 ± 0.8	+36.98 ± 1.1	+35.18 ± 0.5

### Biodistribution

In the lungs, the organ affected by the tumor, the accumulation of MTX by the nanocarrier formulation was significantly higher than that of the conventional powder formulation (*p* < 0.05). Post hoc analysis further revealed a significant MTX accumulation of NFM in tumor tissue compared to NF PI. However, the accumulation in the liver was significantly higher with NFM. On the other hand, the concentrations in the spleen, kidney, and heart did not differ significantly among the groups (Table 6).

**TABLE 6:** Biodistribution studies.

Organ	Marketed PI	NF	NFM
Lung **(**µg)	1.45 ± 0.25	2.04 ± 0.27^a^	2.73 ± 0.19^a^
Liver **(**µg)	16.69 ± 0.20	18.34 ± 1.75	22.94 ± 0.81^a^
Spleen **(**µg)	2.97 ± 0.56	3.26 ± 1.01	3.94 ± 0.91
Kidney **(**µg)	1.79 ± 0.31	2.40 ± 0.87	2.29 ± 0.26
Heart **(**µg)	1.66 ± 0.65	1.52 ± 0.52	1.57 ± 0.45

Data was represented as Mean ± SD (n = 6).

^a^ = (*p* < 0.05); PI = powder for injection; NF , Nanoformulation without lyoprotectant; NFM , nanoformulation with mannitol.

## Discussion

### Lyophilization and particle size

During freeze drying, nucleation begins by drawing the particles together, tending to align in a crystalline form, which generates more opportunities for aggregate formation. Disruption of the nanocarrier in NF could be due to lyophilization-induced stress and the absence of a lyoprotectant, which increased the size enlargement through particle fusion (Wang et al., 2009). Furthermore, the enlargement of particle size beyond the cutoff of 200 nm has raised doubts about the effective EPR targeting ability (Teja and Damodharan, 2018).

It has been predominantly hypothesized that sucrose acts as a lyoprotectant through the vitrification process, forming a thick, viscous layer around nanocarriers that requires high activation energy for sublimation (Wang et al., 2009; Patel et al., 2017). In this context, the observed melt-back phenomenon could possibly be attributed to the inability to achieve very high activation energy, resulting in the formation of a stagnant, viscous layer of sucrose around the nanocarriers, which led to the melt-back of the cake. The melt-back phenomenon is considered a critical stability concern by the FDA and is generally deemed unacceptable according to pharmaceutical standards. Due to these considerations, the NFS formulation was abandoned for further characterization and biodistribution studies.

With regard to the NFM lyophile, the use of mannitol resulted in the formation of a viscous pseudo-hydrated shielding layer around particles during freezing, thereby reducing the extent of their aggregation. Additionally, the absence of internal hydrogen bonding in mannitol increased the likelihood of forming new hydrogen bonds with the individual nanocarriers. This supported the retention of their structure and inhibited particle size enlargement under stress conditions (Wang et al., 2009; Marie et al., 2013).

### Characterization and topographical evaluation of lyophile

Considering the stress induced by lyophilization, the structural examination of the API and excipients emerges as a key parameter in establishing the pharmacological activity and stability of the formulation. The retention of MTX functional groups ensured the integrity of the nanocarriers. However, the establishment of stable polymorphic transformations of mannitol in NFM is crucial, as the conversion to mannitol hemihydrate diminishes product stability, whereas the presence of β-mannitol and δ-mannitol is highly beneficial. XRD examination revealed high-intensity peaks, indicating the presence of anhydrous crystalline polymorphs of mannitol. Furthermore, the absence of diffraction peaks at 16.5° and 180°, which are characteristic signs of mannitol hemihydrate, was observed (Xie et al., 2008). The transformation of mannitol into its polymorphic forms during lyophilization has also been reported by other investigators (Romero-Torres et al., 2007; Xie et al., 2008; Marie et al., 2013). The crystallization of the solute at temperatures below 100°C during freezing controlled the formation of MHH, while drying temperatures above 0°C facilitated the formation of non-hygroscopic crystalline polymorphs of mannitol (Marie et al., 2013).

The nanocarrier aggregation of NF, as supported by SEM analysis, strongly corroborates the theory of aggregation during the nucleation process and the enlargement of particle size. Studies have reported that mannitol exists as thin sheets in its β and δ polymeric forms (Sheskey Paul et al., 2017), thus solidifying the presence of mannitol in its crystalline form without transforming into MHH in the NFM.

### Dissolution and drug release kinetics

As the tumor cellular environment is acidic, pH-mediated drug release of nanocarriers is crucial in safeguarding healthy cells. The elevated drug release in a low pH medium supports the hypothesis of targeting the acidic environment of the tumor through the pH-sensitive behavior of chitosan. Despite the limited solubility of chitosan at pH 7.2, the observed drug release can be attributed to reduced stability in the main and side chains of the polymer due to the amalgamation of heteroatoms during nanocarrier formation. Chitosan, being highly prone to hydrolysis and depolymerization in acidic conditions, resulted in a higher drug release in acidic conditions (Kasaai et al., 2013; Szymańska and Winnicka, 2015). The Korsmeyer-Peppas model and the non-Fickian type endorse the biphasic drug release and hydrolytic behavior of the chitosan polymer, respectively. The higher n-value at pH 5.8 (n = 0.87) clearly indicates that the drug release predominantly relies on the nature of the polymer and supports the pH-responsive drug release of chitosan (Ravichandran, 2013; Pourtalebi Jahromi et al., 2020).

### Product evaluation and stability studies

The lyophilized products comply with the FDA-approved specifications for PI (Pharmaceutical Press, 2016). The pale yellow color of the NF could be attributed to the presence of MTX, while the relatively larger amount of mannitol contributes to the white color of NFM (Jang et al., 2019). The characteristic emissions pertaining to C, N, and P are caused by electron movement from the Kα shell of the respective atoms (Machoy et al., 2016). Additionally, the presence of phosphorus in the sample confirms the effective crosslinking of TPP. Furthermore, particle size analysis and PDI results indicate that particle aggregation and agglomeration did not occur during the study period (Hafner et al., 2011).

### Biodistribution

Tumor vasculature differs from healthy tissue in having fenestrated blood vessels, and the accumulation of nanocarriers in tumor tissues is a key determinant of successful targeting. The significant accumulation of NFM in tumor tissue could be attributed to its smaller particle size, which facilitated the beneficial EPR effect and supported the hypothesis of passive tumor targeting through EPR by smaller particle size (Zubareva et al., 2014). The larger particle size and broader distribution of NF resulted in relatively less accumulation in tumor tissue. In the chaotic blood flow within tumor vasculature, the positive charge of nanocarriers could have been beneficial in reaching the target organ (Blanco et al., 2015). On the other hand, the concentration of MTX observed in healthy organs was lower, which can be attributed to the acidic pH in the tumor region and subsequent drug release. The relatively higher levels of MTX accumulation in the liver and spleen by nanocarriers can be attributed to uptake by phagocytic cells, which has also been observed in other investigations (Zubareva et al., 2014; Ait Bachir et al., 2018). Additionally, the relatively higher accumulation of NF in the kidneys may be an elimination response by the host (Blanco et al., 2015).

## Conclusion

The addition of mannitol as a lyoprotectant proved to be effective in reducing nanocarrier aggregation. However, the effectiveness of sucrose as a lyoprotectant for nanocarriers, particularly chitosan nanocarriers, was found to be ineffective and requires further investigation. The drug release kinetics revealed the dominant role of the polymer, chitosan, as a key determinant in pH-mediated release. The significant increase in MTX concentration in tumor tissue may be attributed to the smaller particle size and surface charge, but further investigation is needed to confirm this. The study demonstrates the potential of chitosan nanocarriers as a tumor-targeting delivery system, enabling site-mediated drug release and potentially reducing the drawbacks associated with standard chemotherapy for improved cancer treatment. Additionally, focusing on the particle dynamics related to size and charge in tumor vasculature is important for successful tumor targeting.

## Data Availability

The raw data supporting the conclusion of this article will be made available by the authors, without undue reservation.

## Abbreviations

CsNP, Chitosan nanocarriers; EPR, Enhanced permeation and Retention; EDS, Energy dispersive spectroscopy; FDA, Food and Drug Administration; FTIR, Fourier transmission infra-red spectroscopy; HPLC, High Performance Liquid Chromatography; ICH, International Conference for harmonization; MTX, Methotrexate; NF, Nanoformulation lyophilized without lyoprotectant; NFS, Nanoformulation lyophilized with sucrose; NFM, Nanoformulation lyophilized with mannitol; PI, Powder for injection; PDI, Poly Dispersity Index; SEM, Scanning electron microscopy; USP, Unites States Pharmacopeia; XRD, X-ray diffraction.

## References

[B1] Ait Bachir Z. Huang Y. He M. Huang L. Hou X. Chen R. (2018). Effects of PEG surface density and chain length on the pharmacokinetics and biodistribution of methotrexate-loaded chitosan nanoparticles. Int. J. Nanomedicine 13, 5657–5671. 10.2147/IJN.S167443 30288039 PMC6161721

[B2] Ball R. L. Bajaj P. Whitehead K. A. (2016). Achieving long-term stability of lipid nanoparticles: Examining the effect of pH, temperature, and lyophilization. Int. J. Nanomedicine 12, 305–315. 10.2147/IJN.S123062 28115848 PMC5221800

[B3] Bhat A. A. Thapa R. Goyal A. Subramaniyan V. Kumar D. Gupta S. (2023). Curcumin-based nanoformulations as an emerging therapeutic strategy for inflammatory lung diseases. Future Med. Chem. 15 (7), 583–586. 10.4155/fmc-2023-0048 37140132

[B4] Blanco E. Shen H. Ferrari M. (2015). Principles of nanoparticle design for overcoming biological barriers to drug delivery. Nat. Biotechnol. 33 (9), 941–951. 10.1038/nbt.3330 26348965 PMC4978509

[B5] Din F. U. Aman W. Ullah I. Qureshi O. S. Mustapha O. Shafique S. (2017). Effective use of nanocarriers as drug delivery systems for the treatment of selected tumors. Int. J. Nanomedicine 12, 7291–7309. 10.2147/IJN.S146315 29042776 PMC5634382

[B6] Duru C. Swann C. Dunleavy U. Mulloy B. Matejtschuk P. (2015). The importance of formulation in the successful lyophilization of influenza reference materials. Biologicals 43 (2), 110–116. 10.1016/j.biologicals.2014.12.001 25614372

[B7] Gangjun D. Guang H. Shuo Z. Haihong L. Mei W. Ji L. (2010). Baicalin suppresses lung carcinoma and lung metastasis by SOD mimic and HIF-1. inhibition. Eur. J. Pharmacol. 630 (1), 121–130. 10.1016/j.ejphar.2009.12.014 20036231

[B8] Golombek S. K. May J. N. Theek B. Appold L. Drude N. Kiessling F. (2018). Tumor targeting via EPR: Strategies to enhance patient responses. Adv. Drug Deliv. Rev. 130, 17–38. 10.1016/j.addr.2018.07.007 30009886 PMC6130746

[B9] Guimarães D. Noro J. Silva C. Cavaco-Paulo A. Nogueira E. (2019). Protective effect of saccharides on freeze-dried liposomes encapsulating drugs. Front. Bioeng. Biotechnol. 7, 424. 10.3389/fbioe.2019.00424 31921827 PMC6927910

[B10] Guzzinati G. Altantzis T. Batuk M. De Backer A. Lumbeeck G. Samaee V. (2018). Recent advances in transmission electron microscopy for materials science at the EMAT lab of the university of antwerp. Mater. (Basel) 11 (8), 1304. 10.3390/ma11081304 PMC6117696 30060556

[B11] Hafner A. Dürrigl M. Pepić I. Filipović-Grčić J. (2011). Short- and long-term stability of lyophilised melatonin-loaded lecithin/chitosan nanoparticles. Chem. Pharm. Bull. (Tokyo) 59 (9), 1117–1123. 10.1248/cpb.59.1117 21881255

[B12] Jang J. H. Jeong S. H. Lee Y. B. (2019). Preparation and *in vitro*/*in vivo* characterization of polymeric nanoparticles containing methotrexate to improve lymphatic delivery. Int. J. Mol. Sci. 20 (13), 3312. 10.3390/ijms20133312 31284483 PMC6651109

[B13] Kasaai M. R. Arul J. Charlet G. (2013). Fragmentation of chitosan by acids. ScientificWorldJournal 2013, 1–11. 10.1155/2013/508540 PMC3835904 24302858

[B14] Krock B. L. Skuli N. Simon M. C. (2011). Hypoxia-induced angiogenesis: Good and evil. Genes. Cancer 2 (12), 1117–1133. 10.1177/1947601911423654 22866203 PMC3411127

[B15] Li J. Cai C. Li J. Li J. Li J. Sun T. (2018). Chitosan-based nanomaterials for drug delivery. Molecules 23 (10), 2661. 10.3390/molecules23102661 30332830 PMC6222903

[B16] Machoy M. Seeliger J. Lipski M. Wójcicka A. Gedrange T. Woźniak K. (2016). SEM-EDS-Based elemental identification on the enamel surface after the completion of orthodontic treatment: *In vitro* studies. Biomed. Res. Int. 2016, 1–5. 10.1155/2016/7280535 PMC5059521 27766265

[B17] Marie H. Larsen L. Trnka H. Grohganz H. (2013). Formation of mannitol hemihydrate in freeze-dried protein formulations—a design of experiment approach. Int. J. Pharm. 1–8. 10.1080/01425692.2013.816041 24239581

[B18] Ngamcherdtrakul W. Sangvanich T. Reda M. Gu S. Bejan D. Yantasee W. (2018). Lyophilization and stability of antibody-conjugated mesoporous silica nanoparticle with cationic polymer and PEG for siRNA delivery. Int. J. Nanomedicine 13, 4015–4027. 10.2147/IJN.S164393 30022824 PMC6045907

[B19] Patel G. Chougule M. Singh M. Misra A. (2009). Chapter 9 - nanoliposomal dry powder formulations. Methods Enzymol. 464, 167–191. 10.1016/S0076-6879(09)64009-X 19903555 PMC3766362

[B20] Patel S. M. Nail S. L. Pikal M. J. Geidobler R. Winter G. Hawe A. (2017). Lyophilized drug product cake appearance: What is acceptable? J. Pharm. Sci. 106 (7), 1706–1721. 10.1016/j.xphs.2017.03.014 28341598

[B21] Pérez-Herrero E. Fernández-Medarde A. (2015). Advanced targeted therapies in cancer: Drug nanocarriers, the future of chemotherapy. Eur. J. Pharm. Biopharm. 93, 52–79. 10.1016/j.ejpb.2015.03.018 25813885

[B22] Pharmaceutical Press (2016). “Injection and implanted drug products (parenterals)-Product Quality Tests,” in Revision bulletin. (London: Pharmaceutical Press). Avaialble at: https://www.uspnf.com/sites/default/files/usp_pdf/EN/USPNF/revisions/gc_1_rb_notice.pdf .

[B23] Pilipenko I. Korzhikov-Vlakh V. Sharoyko V. Zhang N. Schäfer-Korting M. Rühl E. (2019). pH-sensitive chitosan-heparin nanoparticles for effective delivery of genetic drugs into epithelial cells. Pharmaceutics 11 (7), 317. 10.3390/pharmaceutics11070317 31284414 PMC6680926

[B24] Pourtalebi Jahromi L. Ghazali M. Ashrafi H. Azadi A. (2020). A comparison of models for the analysis of the kinetics of drug release from PLGA-based nanoparticles. Heliyon 6 (2), e03451. 10.1016/j.heliyon.2020.e03451 32140583 PMC7049635

[B25] Ravi Kumar M. N. (2000). Nano and microparticles as controlled drug delivery devices. J. Pharm. Pharm. Sci. 3 (2), 234–258. 10.1016/S1381-5148(00)00038-9 10994037

[B26] Ravichandran R. (2013). Studies on dissolution behaviour of nanoparticulate curcumin formulation. Adv. Nanoparticles 2, 51–59. 10.4236/anp.2013.21010

[B27] Romero-Torres S. Wikström H. Grant E. R. Taylor L. S. (2007). Monitoring of mannitol phase behavior during freeze-drying using non-invasive Raman spectroscopy. PDA J. Pharm. Sci. Technol. 61 (2), 131–145. 17479721

[B28] Scimeca M. Bischetti S. Lamsira H. K. Bonfiglio R. Bonanno E. (2018). Energy dispersive X-ray (edx) microanalysis: A powerful tool in biomedical research and diagnosis. Eur. J. Histochem 62 (1), 2841. 10.4081/ejh.2018.2841 29569878 PMC5907194

[B29] Sheskey Paul J. Cook Walter G. Cable Colin G. (2017). Handbook of pharmaceutical excipients. London: Pharmaceutical Press.

[B30] Sreekumar S. Goycoolea F. M. Moerschbacher B. M. Rivera-Rodriguez G. R. (2018). Parameters influencing the size of chitosan-TPP nano- and microparticles. Sci. Rep. 8 (1), 4695. 10.1038/s41598-018-23064-4 29549295 PMC5856823

[B31] Subramaniyan V. Fuloria S. Gupta G. Kumar D. H. Sekar M. Sathasivam K. V. (2022). A review on epidermal growth factor receptor's role in breast and non-small cell lung cancer. Chem. Biol. Interact. 351, 109735. 10.1016/j.cbi.2021.109735 34742684

[B32] Szymańska E. Winnicka K. (2015). Stability of chitosan-a challenge for pharmaceutical and biomedical applications. Mar. Drugs 13 (4), 1819–1846. 10.3390/md13041819 25837983 PMC4413189

[B33] Teja S. P. S. Damodharan N. (2018). 2^3^ full factorial model for particle size optimization of methotrexate loaded chitosan nanocarriers: A design of experiments (DoE) approach. Biomed. Res. Int. 2018, 1–9. 10.1155/2018/7834159 PMC6176313 30356374

[B34] Wang B. Tchessalov S. Cicerone M. T. Warne N. W. Pikal M. J. (2009). Impact of sucrose level on storage stability of proteins in freeze-dried solids: II. Correlation of aggregation rate with protein structure and molecular mobility**This work is a product of the U.S. Government and is not subject to copyright in the United States. J. Pharm. Sci. 98 (9), 3145–3166. 10.1002/jps.21622 19067392

[B35] Xie Y. Cao W. Krishnan S. Lin H. Cauchon N. (2008). Characterization of mannitol polymorphic forms in lyophilized protein formulations using a multivariate curve resolution (MCR)-based Raman spectroscopic method. Pharm. Res. 25 (10), 2292–2301. 10.1007/s11095-008-9624-1 18523875

[B36] Yap K. M. Sekar M. Fuloria S. Wu Y. S. Gan S. H. Mat Rani N. N. I. (2021). Drug delivery of natural products through nanocarriers for effective breast cancer therapy: A comprehensive review of literature. Int. J. Nanomedicine 16, 7891–7941. 10.2147/IJN.S328135 34880614 PMC8648329

[B37] Zubareva A. Shcherbinina T. S. Varlamov V. P. Svirshchevskaya V. (2014). Bio distribution of doxorubicin-loaded succinoyl chitosan nanoparticles in mice injected via intravenous or intranasal routes. Prog. Chem. Appl. Chitin Deriv. 19, 145–154. 10.15259/pcacd.19.18

